# A national virtual job search series for neonatal-perinatal medicine fellows

**DOI:** 10.1186/s12909-024-05587-9

**Published:** 2024-06-06

**Authors:** Nicolle F. Dyess, Brianna Liberio, Sarah Bernstein, Sharla Rent, Heather French, Patrick Myers

**Affiliations:** 1 Department of Pediatrics, Section of Neonatology, University of Colorado, 13121 East 17th Avenue, Mail Stop 8402, Room 4304, Aurora, Colorado 80045 USA; 2 Department of Pediatrics, Division of Neonatal-Perinatal Medicine, Indiana University, Indianapolis, Indiana USA; 3 Department of Pediatrics, Section of Neonatology, The University of Utah, Salt Lake City, Utah USA; 4 Department of Pediatrics, Section of Neonatology, Duke University, Durham, North Carolina USA; 5 Department of Pediatrics, Section of Neonatology, The Children’s Hospital of Philadelphia, Perelman School of Medicine at the University of Pennsylvania, Philadelphia, USA; 6 Department of Pediatrics, Section of Neonatology, Feinberg School of Medicine, Northwestern University, Chicago, Illinois USA

**Keywords:** Career development, Career transition, Fellowship, Job search, Neonatology, Trainee

## Abstract

**Background:**

A standardized approach to prepare trainees for the job search has not been described. The objective of this study was to describe and evaluate an educational series on the job search for Neonatal-Perinatal Medicine (NPM) fellows and identify participants’ job search knowledge gaps.

**Methods:**

During the 2020–2021 academic year, we created a virtual, seven-part job search series for NPM fellows that required no funding. The series has been repeated annually. We use REDCap surveys to register participants, collect baseline/demographic information, and evaluate the series’ impact at the beginning and end of the job search timeline.

**Results:**

In the 2021–2022 academic year, 290 individuals registered for the series, and 89% completed the baseline/demographic survey. The majority were NPM fellows (89%). Early career neonatologists, NPM hospitalists, and pediatric residents also utilized the series (11%). Less than 25% reported being “knowledgeable” or “very knowledgeable” of core job search components, including the timeline of the job search, contract negotiation, and the general roles and responsibilities of junior faculty. Of those who completed the final job search survey and underwent a job search (60%, 97 of 162), the majority (86%) felt that career planning during training was stressful and believed that job search preparation should be structured into the NPM fellowship curriculum (81%). Many felt that the Job Search Series was helpful in elucidating components of the job search.

**Conclusions:**

We identified several knowledge gaps in NPM fellows’ understanding of how to find, prepare for, and negotiate their first post-training job. We strongly believe these knowledge gaps are not unique to NPM fellows and that all graduate medical education trainees would benefit from a similar, easy-to-implement, no-cost series.

**Supplementary Information:**

The online version contains supplementary material available at 10.1186/s12909-024-05587-9.

## Introduction

A standardized approach to preparing medical trainees for their first job search has not been described in the literature. For the entirety of their education and training, trainees pursuing a sub-specialty career follow a relatively standard roadmap of distinct stages with well-defined processes that govern the transition from one stage to another. However, as formal education and training programs near conclusion, medical trainees are tasked with finding their first job, a process which is unfamiliar, ill-defined, and unlike any prior stage, often leaving trainees feeling anxious and ill-equipped for this major professional transition. Although many aspects of the job search may be unique to a particular trainee, many commonalities exist, providing the opportunity for a standardized approach to prepare trainees for their first post-training job search.

In a study of career planning among graduating internal medicine residents, 75% of trainees found career planning at least somewhat stressful [1]. Few trainees have access to centralized resources or receive formal guidance from their mentors or training program leadership to prepare for the job search [1, 2]. Trainees desire formal training regarding the job search and interview processes [2] and have previously identified five knowledge areas that should be integrated into training curricula: (1) the job search process, (2) career paths and opportunities, (3) anticipated conflicting timelines and responsibilities, (4) importance of mentorship, and (5) importance of self-reflection regarding priorities and desired outcomes [1]. 

There have been increasing reports of strategies to address the need for centralized resources and courses to prepare trainees for the job search. Several online resources, personal reflections, and descriptive papers now exist to guide trainees and program directors (PDs) [3–12]. Some have published curricula [13–16] or career panels [17, 18] implemented at single institutions concerning individual components of the job search. However, to our knowledge, an attempt to provide comprehensive, national education on the job search process has not been described in the graduate medical education (GME) literature.

Before the Covid-19 pandemic, neonatal-perinatal medicine (NPM) fellows identified job openings through word of mouth, emailing division chiefs/medical directors, networking at conferences, and/or using recruiters. Interviews were often in person, requiring NPM fellows to request time off from their fellowship programs for travel. The advent of the COVID-19 pandemic unfortunately aligned with the final stages of the graduating NPM fellow hiring cycle in the spring of 2020. During this time, the American Academy of Pediatrics’ (AAP) Organization of Neonatal-Perinatal Medicine Training Program Directors (ONTPD) held multiple meetings to share resources, build virtual curricula [19, 20], and prepare a unified response to the Accreditation Council of GME (ACGME). It became apparent that the COVID-19 pandemic was leading to a changing job market for graduating NPM fellows. NPM PDs and fellows reported having job offers altered or withdrawn and interviews canceled. This phenomenon occurred across the GME community [21] and many medical fields [22]. Many PDs reported COVID-19 anxiety in their trainees [23], with graduating fellows having anxiety centered around their job and career prospects. Job search anxiety continues to be reported by PDs in ONTPD virtual cafés and breakout sessions at national meetings.

In response, ONTPD, in collaboration with the AAP’s Trainees and Early Career Neonatologists (TECaN) special interest group, created a formal, virtual curriculum to prepare trainees across the United States (US) for the transition to their first post-training jobs and what to expect during the job search process. We predicted trainees would benefit from the experiences of recent graduates and senior-level neonatologists on navigating the job market and that this program would ultimately lead to a more informed and less stressful job search. This report describes and evaluates the ONTPD and TECaN National Virtual Job Search Series for NPM fellows and identifies participants’ job search knowledge gaps.

## Methods

A virtual, seven-part job search series curriculum was developed to provide guidance and reduce anxiety about job hunting for NPM fellows following Kern’s model of curriculum development [24]. Because fellows’ needs were perceived to be urgent, the curriculum was developed rapidly. An evaluation of the literature identified significant knowledge gaps on best practices in the fellowship job search research resulting in a focus on problem identification/general needs assessment (Kern’s step 1). This was performed through a series of meetings with PDs, fellows, and section chiefs to determine curricular goals, session learning objectives, and educational strategies (Kern’s steps 3 and 4). The curriculum was then piloted during the 2020–2021 academic year (Kern’s step 5). Since its development, the series has been repeated annually. During the 2021–2022 academic year, a targeted needs assessment (Kern’s step 2) and curriculum evaluation (Kern’s step 6) was completed. We discovered that early career neonatologists, NPM hospitalists, and pediatric residents were also registering for and utilizing the series, in addition to NPM fellows, an outcome we had not anticipated. As such, we included these populations in our targeted needs assessment. Ongoing general needs assessments and evaluations have occurred as the series progressed. The results of our 2021–2022 targeted needs assessment and curriculum evaluation are reported here.

The series’ sessions included didactics on the timeline and expectations of the job search, the experiences of applicants underrepresented in medicine, gender pay inequity, and job search fundamentals. Additionally, multiple career panels with practicing neonatologists from both academic and community medicine were held (Table 1). Didactic and panel objectives and affective learning objectives used to evaluate the curriculum are depicted in Additional File 1. ONTPD and TECaN co-hosted the one-hour sessions on Zoom (Zoom Video Communication, Inc.). As NPM fellowship programs span various time zones, we chose to start the sessions at 3PM Eastern to minimize conflict with patient care activities (i.e., morning rounds, afternoon sign out, etc.). For those unable to make the live session, a link to the recorded session was sent to all registrants. Furthermore, the recorded sessions were publicly available on the TECaN and ONTPD YouTube channels.

**Table 1:** Composition of national virtual job search series during the 2021–2022 academic year

Session	Format	Description	Number of Registrants
1	Didactic & Panel	Overview Didactic: Interviewing overview: preparing your curriculum vitae and cover letter; marketing yourself; the interview season roadmap; virtual interview best practices; time, salary, professional identity formation, and benchmark data for early career neonatologists (3 speakers) Diversity, Equity, and Inclusion (DEI) Career Panel: Tailored Q&A for underrepresented in medicine trainees and those with career interests in DEI (2 panelists)	229
2	Panel	Junior Faculty Panel: Transition to attending life, the job search, and Zoom interviewing (6 panelists)	223
3	Panel	Academic Section Chiefs: Interview tips and advice for pursuing an academic career (4 panelists)	218
4	Didactic & Panel	Visa Applicants: Tailored didactic (2 speakers) and Q&A for trainees on a visa (3 foreign medical graduate junior faculty panelists)	227
5	Panel	Private Practice: Guidance from senior and junior attending physicians in private practice on the job search and transition from fellowship (4 panelists)	105
6	Didactic & Panel	Negotiations Didactic: Managing job negotiations, multiple job offers, and making a final decision (2 speakers) Didactic and Q&A: Contract negotiation for women to help reduce gender inequality in starting salary (1 speaker and 3 panelists)	254

To enhance their professional networks, NPM fellows were involved with organizing and moderating these sessions. An open call for moderators was advertised through TECaN’s listserv and encrypted instant messaging groups (Signal Messenger LLC), and fellow moderators were chosen based on their availability, interest, and expertise for the sessions and post-training career plans. Sessions were advertised through social media platforms, website postings, and sub-specialty listservs.

No funding was needed to implement the series. Our Job Search Series planning committee consisted of two NPM fellows, two junior faculty neonatologists who were members of the TECaN executive council, and two senior faculty neonatologists who were members of the ONTPD executive board. For the career panels, the committee utilized professional networks to recruit a diverse group of speakers consisting of recently graduated NPM fellows, private practice physicians from a wide range of practice models, neonatologists from underrepresented in medicine backgrounds, and academic section chiefs. Time commitments, divided among committee members, included recruitment of panel members (~ 2 h); development of registration survey and end of job search survey (~ 10 h); identifying moderators (~ 4 h); collating questions for panelists (~ 7 h); advertising the sessions (~ 7 h); and distributing Zoom chat texts, links to recorded sessions, and follow-up questions and answers to registrants (~ 10 h). Time commitments decreased in subsequent years.

Individuals registered and submitted questions for the sessions via Research Electronic Data Capture (REDCap) [25, 26] survey (Additional File 2). Registrants could enroll for a subset of the sessions of their choosing or for the entire series. Demographic information and baseline job search knowledge were collected through the registration survey, and registrants could submit questions to be answered by the speakers and panelists during the live sessions. The individual Zoom links for each session were sent to everyone who registered for that session.

Near the end of the 2021–2022 academic year, five survey reminders were sent to disseminate the final job search survey (Additional File 2). A portion of this survey collected data on the utility of the series, feedback to improve the series, topics of interest for future series, and whether the fellow was successful or not in obtaining a job, which was a key outcome of our curriculum.

We followed a systematic approach to survey design [27] when constructing our survey instruments to optimize quality, reliability, and validity. Survey questions were constructed in alignment with established best practices [28–30]. Medical education leaders within ONTPD pretested the survey instruments for clarity and face validity via cognitive interviews. We piloted the survey on TECaN’s executive council (*n* = 18) which was comprised of current NPM fellows and junior faculty neonatologists who recently underwent the job search. Although emails were used for registration, survey data was analyzed after emails were removed, ensuring anonymity.

We used descriptive statistics (i.e., counts and frequencies) to analyze quantitative survey data. There were three free-response survey questions within our targeted needs assessment: (1) What have you found most challenging about your job search? (2) What did you wish you had known prior to your job search? and (3) What tools or topics regarding the job search would be helpful for future trainees? We used descriptive qualitative analysis to identify categories of answers to the surveys’ free-text responses [31]. 

All methods were performed in accordance with the Declaration of Helsinki, study was approved by the ethics committee of the University of Colorado’s Institutional Review Board and deemed exempt as a program evaluation (#21-3214). The study was described on the welcoming page of each survey, and by completing the survey(s), registrants of the series provided their informed consent to participate in this study.

## Results

During the 2021–2022 academic year, 290 individuals registered for the series, with 53% undergoing a job search that year (Table 2). Those not undergoing the job search included NPM fellows in the first two years of fellowship and pediatric residents. Registrants represented 35 states and 75% (82/110) of the NPM fellowship programs in the US. Of the NPM fellowship programs not represented, 32% (9/28) did not yet have any senior fellows during the 2021–2022 academic year.

**Table 2:** Characteristics of job search series registrants

Characteristic (total number of responses possible)	N (%)		
Entering the job search (*N* = 290)			
Yes	154 (53)		
No	111 (38)		
Unsure	25 (9)		
Completed demographic survey (*N* = 290)			
Yes	257 (89)		
No	33 (11)		
Gender Identification (*N* = 257)			
Male	60 (23)		
Female	193 (75)		
Prefer not to disclose	1 (1)		
Missing	3 (1)		
Ethnicity^a^ (*N* = 257)			
White	132		
Hispanic/Latinx	25		
Black/African American	9		
Native American/American Indian	1		
Asian/Pacific Islander	76		
Other	11		
Prefer not to disclose	12		
NPM fellow (*N* = 257)			
Yes	228 (89)		
No	25 (10)		
Missing	4 (1)		
Job type desires (*N* = 257)^a^			
Academic	181		
Private practice	138		
Mixed model	147		
Military	0		
Locums	16		
Other	1		
Unsure	1		
If academics, desired level of research commitment (*N* = 181)			
Mainly research	16 (9)		
Some scholarly expectation	110 (61)		
100% clinical	18 (10)		
Undecided	36 (20)		
Other	1 (1)		

^a^ Question was “choose all that apply,” thus could not calculate percentage

**Fig. 1:**
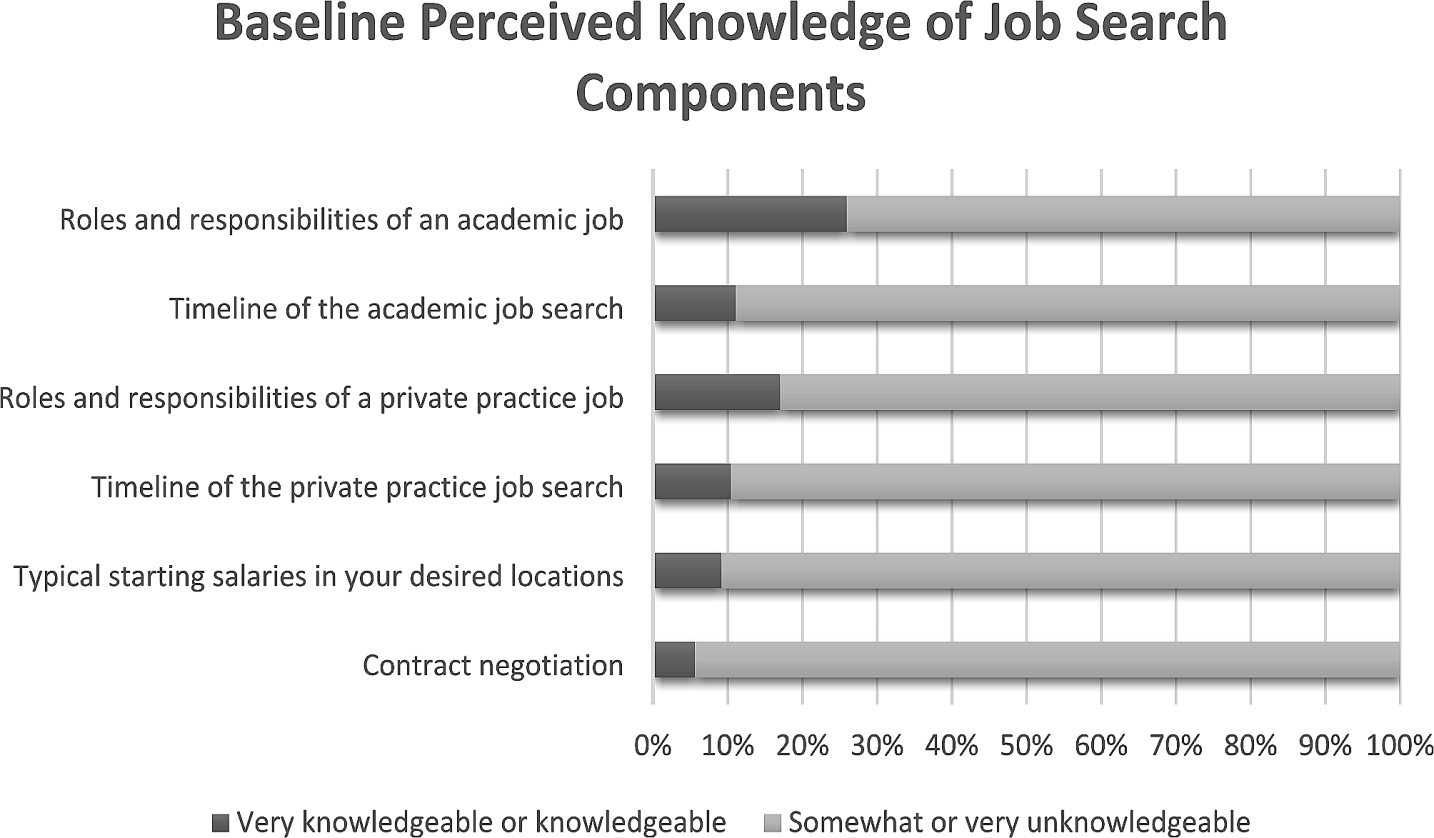
– Baseline Perceived Knowledge of Job Search Components at Time of Job Search Series Registration

**Fig. 2:**
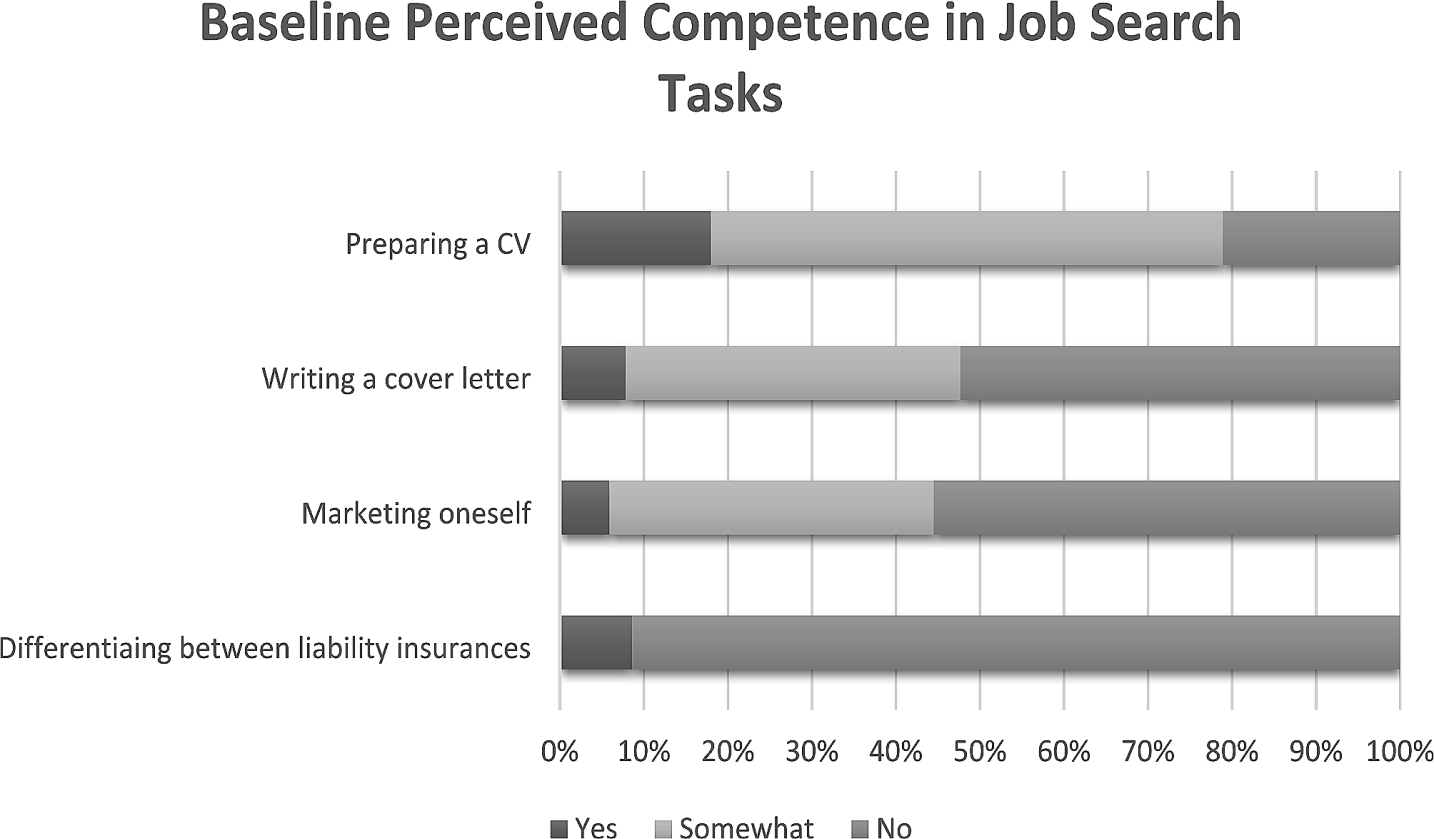
Baseline perceived competence in job search tasks at time of job search series registration

Two hundred and fifty-seven registrants (89%) completed the demographics section of the registration survey. 76% of registrants were female and 89% were NPM fellows. Non-NPM fellow registrants included neonatal intensive care unit hospitalists, neonatologists, and pediatric residents. Less than 25% reported being “knowledgeable” or “very knowledgeable” of job search components, such as the general roles and responsibilities of junior faculty, the timeline of the job search, the typical starting salaries in their desired geographic locations, and contract negotiation (Fig. 1). Many registrants did not feel competent writing a cover letter (52%) or marketing themselves (55%) (Fig. 2). 91% lacked basic understanding of malpractice insurance (Fig. 2).

63% of registrants (162/257) who completed the baseline demographic survey also completed the final job search survey at the conclusion of the series. Of those who completed the final survey and ultimately underwent a job search (60%), over half (57%) did not have a clear understanding of the various practice models within neonatology when they entered fellowship. Strategies utilized to gain knowledge of practice models consisted of this job search series, the job application and interview process, and informal discussions with mentors, social contacts, and previous fellows.

**Fig. 3:**
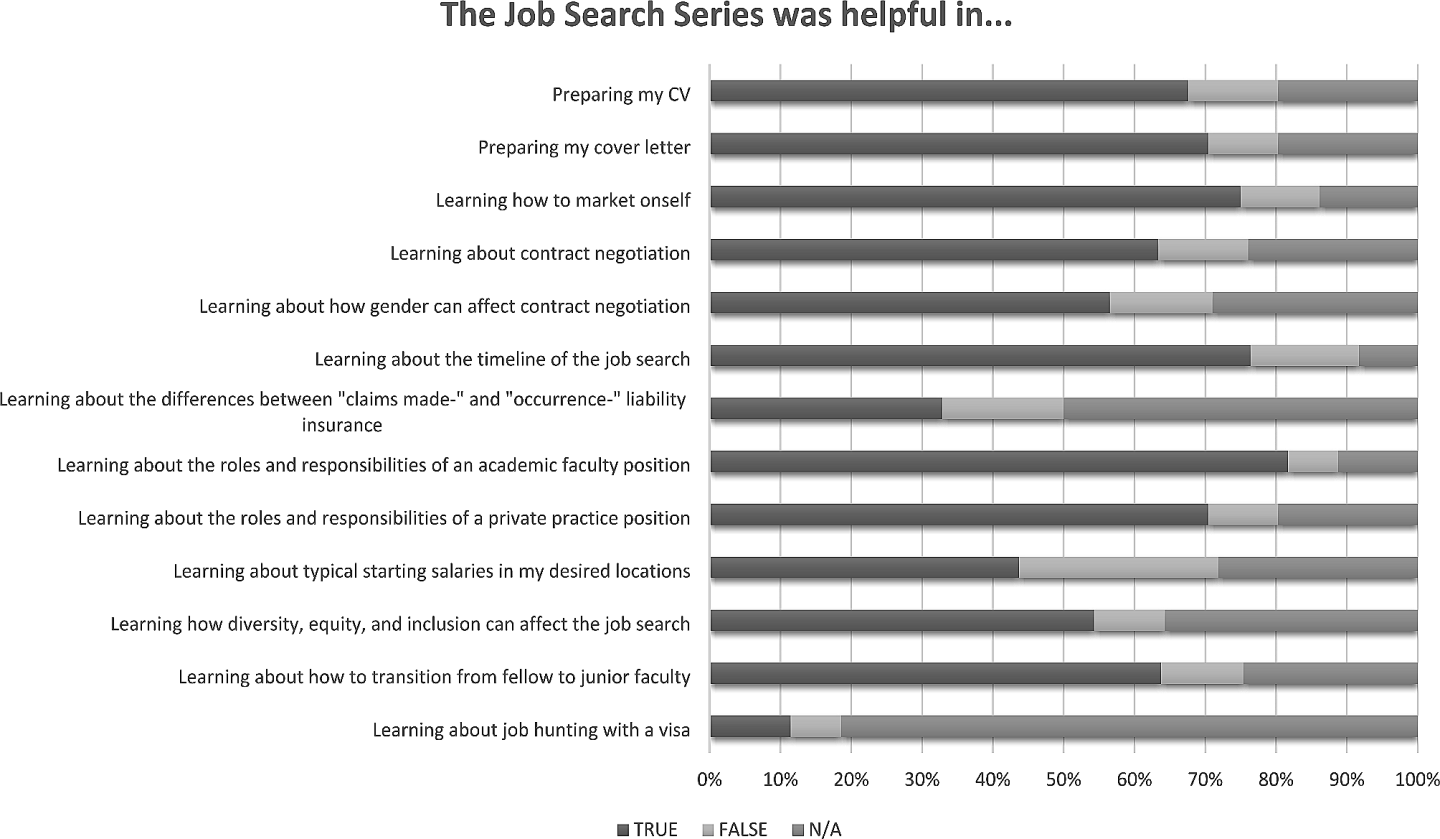
Survey respondent-reported helpfulness of ONTPD/TECaN job search series

Most respondents who underwent a job search during the academic year and completed the final survey felt that career planning during training was stressful (86%). A majority believed that job search preparation should be structured into the NPM fellowship curriculum (81%) and that the Job Search Series was helpful in elucidating many job search components (Fig. 3), accomplishing all our affective learning objectives (Additional File 1). Although 40% of the final survey respondents did not undergo a job search during that academic year, nearly half (48%) still utilized the job search series, with the intent to plan a future job search (81%) and to learn about practice models (71%).

The final survey also served as a needs assessment and method to improve the Job Search Series in subsequent years with free-text questions found in Additional File 3. The top three issues that registrants found challenging were categorized as process issues, personal issues, and program/mentorship issues. The top three answers were navigating the job search timeline (process issue), identifying hiring institutions/practices (process issue), and geographical limitations (personal issue). Registrants had five major areas that they wished they had known more about: the amount of time required by the job search, the volume and frequency of communication from prospective employers, the process of finding available jobs, and the timeline of the job search. In addition, survey respondents recommended topics to improve the series with suggestions categorized by the need for more information on either academic positions, private practice positions, or both.

Seventy-five NPM fellows who participated in the job search during the academic year detailed their individual job search experiences in the final survey. By March 2022, only 7% had not yet received a job offer. Of those who had received a job offer (93%), the majority (86%) had accepted an offer and most (71%) had signed a final contract.

## Discussion

Many NPM fellows are unfamiliar with numerous components of the job search and attending physician practice models available after training. Surveyed fellows perceive the job search process as stressful and believe that job search preparation should be integrated into fellowship curricula. This virtual, cost-free job search series helped close many of the identified knowledge gaps about job search processes and provided a common curriculum that can be utilized by all medical trainees entering the job search. Fellows found ONTPD/TECaN’s Job Search Series instrumental to their job searches and provided insight into challenges which can be used to advise future trainees and revise future iterations of the series.

The job search timeline and the challenges identified by NPM fellows are shared by many medical specialties [2, 3, 21, 32–34]. A study of critical care medicine fellows showed that less than half of trainees receive formal guidance from mentors or their training programs on the job search [2]. Other GME training programs can utilize the ONTPD/TECaN Job Search Series as a model for similar programing for their trainees, especially since the desire to incorporate job search preparation into training curricula is common across specialties [1]. 

The timing of the afternoon live sessions along with the availability of recorded sessions increase the program’s accessibility and have allowed us to reach fellows across the nation. As of February 1^st^, 2024, the Job Search Series has had just over 3,300 views on YouTube. The reach of our Job Search Series continues to improve every year. The 2022–2023 academic year marks the third year we have implemented this series, and as of the end of October 2022, we have had 348 unique registrations (an increase of 58 registrations from the year prior), spanning 37 states, and representing 84% (92/110) of the NPM fellowship programs in the US (up from 75% the year prior). Of the NPM fellowship programs not represented (18/110), eight do not yet have senior fellows.

This program evaluation has several limitations. Academic neonatologists and fellows designed the surveys used to evaluate this curriculum which increases the risk of selection bias in survey development and face validity by not including neonatologists practicing in non-university-based practice models. However, annually, we host a webinar focused on private practice as part of the Job Search Series. Only registrants of the Job Search Series were surveyed, introducing the possibility of selection bias. Those who provided details about their job search experience were subject to recall bias. As there is no published data on the specifics of NPM fellows’ first post-training job searches, we cannot compare if our registrants’ job search results were different than those who did not utilize the series. We hope to survey all graduating fellows in the future to address some of these limitations. Additionally, data analysis occurred in aggregate. In future research, we hope to understand the different needs of the various constituents of the job search series. Learner assessment is also a part of program evaluation. However, learner assessment of a national cohort is challenging, and, thus, this is a limitation of our program evaluation. Lastly, the results of this program evaluation should only be used as a guide, as many factors interplay in an individual fellow’s job search experience and future professional responsibilities and compensation.

We aim to trend this data annually to better serve each upcoming cohort of trainees entering the job search. Using the feedback provided by our registrants, we have dedicated more time to the details of the job search timeline/process and have incorporated some of the recommended topics into subsequent iterations of our Job Search Series such as liability insurance, how to advocate for protected research time, job talk tips, and interview tips. It will also be important to obtain more granular data on other specific components of the job search such as trainee’s understanding of clinical full-time equivalents, revenue value units, and compensation models. As more trainees and junior faculty utilize ONTPD/TECaN’s Job Search Series, we anticipate that our results will become more representative of the graduating class of NPM fellows over time. Additionally, we have started advertising more globally, to include post-training neonatologists. Approximately 10% of our registrants each year have been practicing neonatologists, highlighting a need to understand why post-graduates are attending the series and potentially curate content to meet their unique needs. Finally, following registrants of the Job Search Series longitudinally will be useful to better understand their employment trajectory, as it is common for physicians to switch jobs [35]. 

## Conclusion

We describe a formal, virtual curriculum to prepare trainees across the US for the job search. Curriculum development focused on rectifying the knowledge gaps identified which included an understanding of how to find one’s first attending job, how to prepare for the job search, the different practice models available, and how to negotiate a position. We strongly believe these knowledge gaps are not unique to NPM fellows and that all GME trainees would benefit from a similar, easy-to-implement, no-cost job search curriculum. YouTube views of the series have more than doubled in the last 18 months which indicates that there continues to be desire for this content in the NPM community.

### Electronic supplementary material

Below is the link to the electronic supplementary material.

Supplementary Material 1

Supplementary Material 2

Supplementary Material 3

## Data Availability

All datasets used and/or analyzed during the current study are available from the corresponding author on reasonable request.

## Abbreviations

AAP: American Academy of Pediatrics
ACGME: Accreditation Council of Graduate Medical Education
GME: Graduate Medical Education
NPM: Neonatal-Perinatal Medicine
ONTPD: Organization of Neonatal-Perinatal Medicine Training Program Directors
PD: Program Director
REDCap: Research Electronic Data Capture
TECaN: Trainees and Early Career Neonatologists
US: United States
