# Supplementary material for: A national virtual job search series for neonatal-perinatal medicine fellows
Source: BMC Med Educ. 2024 Jun 6;24:633. doi: 10.1186/s12909-024-05587-9 (PMC11155180; doi:10.1186/s12909-024-05587-9)
Supplement: Supplementary file 1 — Supplementary Material 1 [file 12909_2024_5587_MOESM1_ESM.pdf]

Additional File 1. Curricular Learning Objectives

Job Search Series Session	Learning Objectives
1. Job Search Overview Didactic and Diversity, Equity, and Inclusion Career Panel	Didactic Objectives:<br>* Define the components of a CV<br>* Describe how to best structure a CV<br>* Define the components of a cover letter<br>* Describe how to best structure a cover letter<br>* Understand the importance of self-marketing on the job search<br>* Describe the road map of the interview season<br>* Summarize, compare, and contrast the time, salary, and personal identities of early career neonatologists<br><br>Panel Objective:<br>* Answer questions submitted by registrants regarding how diversity, equity and inclusion can affect the job search<br><br>Learner affective learning objectives:<br>* The majority of learners will state (in the end of the academic year job search survey) that the 1st session of the Job Search Series was helpful in:<br>- preparing their CV<br>- preparing their cover letter<br>- learning how to market themselves<br>- learning about the timeline of the job search<br>- learning the difference between “claims made-“ and “occurrence-“ liability insurance<br>- learning about typical starting salaries in my desired locations<br>- learning how diversity, equity and inclusion can affect the job search
2. Junior Faculty Panel	Panel Objective:<br>* Answer questions submitted by registrants regarding the transition from fellow to jr faculty<br><br>Learner affective learning objectives:<br>* The majority of learners will state (in the end of the academic year job search survey) that the 2nd session of the Job Search Series was helpful in:<br>- learning about the transition from fellow to jr. faculty
3. Academic Section Chief Panel	Panel Objective:<br>* Answer questions submitted by registrants regarding academic job searches

	Learner affective learning objectives:<br>* The majority of learners will state (in the end of the academic year job search survey) that the 3rd session of the Job Search Series was helpful in:<br>- learning about the roles and responsibilities of an academic faculty position
4. Job Hunting with a Visa Didactic and Career Panel	Session Objective:<br>* Describe the job search with different types of VISAs<br>* Describe the intricacies of the J1 waiver<br><br>Panel Objective:<br>* Answer questions submitted by registrants regarding job hunting with a VISA<br><br>Learner affective learning objectives:<br>* The majority of learners will state (in the end of the academic year job search survey) that the 4th session of the Job Search Series was helpful in:<br>- learning about job hunting with a VISA
5. Private Practice Panel	Panel Objective:<br>* Answer questions submitted by registrants regarding private practice job searches<br><br>Learner affective learning objectives:<br>* The majority of learners will state (in the end of the academic year job search survey) that the 5th session of the Job Search Series was helpful in:<br>- learning about the roles and responsibilities of a private practice faculty position
6. Contract Negotiation and Negotiating for Women Didactic and Panel	Didactic Objectives:<br>* Evaluate personal priorities related to first attending level job<br>* Understand the mechanics of the negotiation process<br>* Gain insight into the Chief perspective on negotiating with new graduates<br>* Identify action items to resolve prior to next interview/negotiation<br>* Compare and contrast salaries, leadership, and promotion for women in medicine and neonatology<br><br>Panel Objectives:<br>* Answer questions submitted by registrants regarding contract negotiation<br>* Answer questions submitted by registrants regarding how gender can affect contract negotiation

	Learner affective learning objectives:<br>* The majority of learners will state that the 6th session of the Job Search Series was helpful in:<br>- learning about contract negotiation<br>- learning about how gender can affect contract negotiation
