# Supplementary material for: A national virtual job search series for neonatal-perinatal medicine fellows
Source: BMC Med Educ. 2024 Jun 6;24:633. doi: 10.1186/s12909-024-05587-9 (PMC11155180; doi:10.1186/s12909-024-05587-9)
Supplement: Supplementary file 2 — Supplementary Material 2 [file 12909_2024_5587_MOESM2_ESM.pdf]

Additional File 2: Survey Instruments

ONTPD/TECaN Virtual Job Search Series Registration Survey

Welcome to ONTPD's and TECaN's Virtual Job Search Workshop and Career Panels Survey!

This brief survey is being conducted to understand how neonatology fellows fell about the utility of ONTPD's and TECaN's virtual job search workshop and/or career panels. We are interested in the views of all neonatology fellows who plan to view at least one of these sessions regardless of whether or not they plan to enter the job search this academic year (2021-22). In addition to this survey, there will be a post-session evaluation survey and a short end of the year job search survey.

We hope you will take a moment to complete the survey. Your participation is voluntary and your responses will be kept confidential. No personally identifiable information will be associated with your responses in any reports of the data.

If filling out this survey after completion of the live event, we will send you the recording of the session(s) upon registration completion.

Thank you,

ONTPD and TECaN Virtual Job Search Series Committee

1. Name: ________________
2. Email: ________________
3. Are you planning on searching for a job this upcoming academic year (2021-2022)?
    a. Yes
    b. No
    c. Unsure
4. Have you filled out his registration survey already this year?
    a. Yes → Q5
    b. No → Q22
5. Fellowship Program Name: ____
6. How old are you? ______________________ years
7. What is your gender identification?
    a. Male
    b. Female
    c. Transgender male
    d. Transgender female
    e. Gender variant/non-conforming
    f. Other → Please describe: ___________________________________
    g. Prefer not to answer
8. What is your ethnicity? You may choose more than one response.
    a. White
    b. Hispanic/Latinx
    c. Black/African American

   d. Native American/American Indian
   e. Asian/Pacific Islander
   f. Other → Please describe: ___________________________________
   g. Prefer not to answer
9. What year of fellowship are you in?
   a. 1st
   b. 2nd
   c. 3rd
   d. $\geq 4^{th}$
   e. Other → Please describe: ___________________________________
10. What job type do you ultimately plan to apply to? You may choose more than one response.
   a. Academic → Q11
   b. Private → Q12
   c. Mixed model → Q11
   d. Military → Q12
   e. Locums → Q12
   f. Other → Please describe: ___________________________________
11. If academics, what is your desired level of research commitment?
   a. Mainly research (ie, research track)
   b. Some scholarly expectation (ie, clinical-educator or clinical-scholar track)
   c. 100% clinical
   d. Undecided
   e. Other → Please describe: ___________________________________
12. Do you feel competent in preparing a CV?
   a. Yes
   b. Somewhat
   c. No

   d. No opinion
13. Do you feel competent in writing a cover letter?
   a. Yes
   b. Somewhat
   c. No

   d. No opinion
14. Do you feel competent in marketing yourself?
   a. Yes
   b. Somewhat
   c. No

   d. No opinion

15. How knowledgeable or unknowledgeable do you feel about the timeline of the academic job search?
    a. Very knowledgeable
    b. Knowledgeable
    c. Somewhat unknowledgeable
    d. Very unknowledgeable

    e. No opinion

    f. Not applicable
16. How knowledgeable or unknowledgeable do you feel about the timeline of the private practice job search?
    a. Very knowledgeable
    b. Knowledgeable
    c. Somewhat unknowledgeable
    d. Very unknowledgeable

    e. No opinion

    f. Not applicable
17. How knowledgeable or unknowledgeable do you feel about contract negotiation?
    a. Very knowledgeable
    b. Knowledgeable
    c. Somewhat unknowledgeable
    d. Very unknowledgeable

    e. No opinion

    f. Not applicable
18. How knowledgeable or unknowledgeable do you feel about typical starting job salaries in your desired location(s)?
    a. Very knowledgeable
    b. Knowledgeable
    c. Somewhat unknowledgeable
    d. Very unknowledgeable

    e. No opinion

    f. Not applicable
19. How knowledgeable or unknowledgeable do you feel about the roles and responsibilities of an academic job?
    a. Very knowledgeable
    b. Knowledgeable

c. Somewhat unknowledgeable
d. Very unknowledgeable

e. No opinion

f. Not applicable

20. How knowledgeable or unknowledgeable do you feel about the roles and responsibilities of a private practice job?
    a. Very knowledgeable
    b. Knowledgeable
    c. Somewhat unknowledgeable
    d. Very unknowledgeable

    e. No opinion

    f. Not applicable
21. Do you know the difference between “claims made-“ and “occurrence-“ liability insurance?
    a. Yes
    b. No
22. What session(s) would you be interested in attending / receiving the recorded link for?
    a. May 10th, 3-5PM EST – Didactic #1: Interviewing overview didactic with Q&A + diversity, equity, and inclusion panel (preparing your CV and marketing yourself; the “roadmap” of the interview season; time, salary, and personal identity for early career neonatologists; health care and adjunct staff)
        i. → If chosen, what questions would you like addressed during the Interviewing Overview (didactic #1) session?
    b. May 21st, 3-4PM EST – Junior Faculty Panel: focused on the transition to attending life, the job search, and zoom interviewing
        i. → If chosen, what questions would you like to ask the junior faculty panelists?
    c. June 4th, 3-4PM EST – Academic Section Chief Panel: what academic NICUs are looking for in new faculty, interview tips and advice
        i. → If chosen, what questions would you like to ask the academic section chief panelists?
    d. June 17th, 2-3PM EST – Job Hunting with a Visa: tailored didactic and Q&A for neonatologists with a Visa
        i. → If chosen, what questions would you like addressed during the Job Hunting with a Visa session?
    e. June 18th, 3-4PM EST – Private Practice Panel: guidance from senior and junior neonatologists in private practice on the job search and transition from fellowship
        i. → If chosen, what questions would you like to ask the private practice panelists?

f. October 4th, 3-4PM EST – Didactic #2: managing job negotiations, multiple job offers, and making a final decision + negotiating for women
    i. → If chosen, what questions would you like addressed during the Job Offers and Negotiations (didactic #2) session?

Final Job Search Survey: Email solicitation on 3/1/2021 followed by 4 email reminders (3/7, 3/10, 3/18, and 3/24).

Welcome to ONTPD's and TECaN's Virtual Job Search Series – Final Job Search Survey!

This brief survey is being conducted to better characterize the neonatology job search process. We are interested in the views of all those who registered for the ONTPD/TECaN Job Search Series, regardless of whether or not you utilized this resource and whether or not you completed a job search this year.

This important study will help guide fellows and early career neonatologists entering the job search in upcoming years and will also help refine the ONTPD/TECaN Job Search Series resource.

We hope you will take a moment to complete the survey. Your participation is voluntary and your responses will be kept confidential. No personally identifiable information ill be associated with your responses in any reports of the data.

Thank you,

ONTPD/TECaN Virtual Job Search Series Committee

1. Did you undergo a job search this academic year (2021-2022)?
    a. Yes → Q4
    b. No → Q2
2. Did you utilize the ONTPD/TECaN Job Search Series?
    a. Yes → Q3 and then end survey
    b. No → end survey
3. Why did you participate in the Job Search Series? (check all that apply)
    a. Planning for a future job search
    b. To learn about various practice models
    c. To network
    d. Other → for what other reasons(s) did you participate in the Job Search Series? (free text)
4. When you entered fellowship, did you have a clear understanding of the various practice models (ie, academic, private, etc) that are available to practicing neonatologists?
    a. Yes → Q5
    b. No → how did you gain knowledge of the various practice models available to practicing neonatologists? (free text) then Q6
5. What type of neonatal practice did you intend to join when you entered fellowship?
    a. Academic
    b. Private Practice
    c. Other → what type of Neonatal practice did you intend to join when you entered fellowship?
6. What type of Neonatal practice are you looking for/joining after fellowship?
    a. Academic

b. Private practice
c. Other → what type of neonatal practice are you looking for/joining after fellowship? (free text)
d. Undecided
e. Not entering Neonatal practice → please elaborate (free text)

The next set of questions will help us better describe the general job search process and resources available to future fellows and early career neonatologists entering the job search.

7. When did your fellowship program tell you to start your job search (ie, start drafting your CV and cover letter, start reaching out to programs, etc)
   a. During first year
   b. Early second year
   c. Late second year
   d. Early third year
   e. Late third year
   f. Other → when did you fellowship program tell you to start your job search? (free text)
   g. No recommendation → Q9 then Q11
8. Did you start your job search either before or after the time recommended by your fellowship program?
   a. Yes → Q9
   b. No → Q10
9. When did you start your job search?
   a. During first year
   b. Early second year
   c. Late second year
   d. Early third year
   e. Late third year
   f. Other → when did you start your job search? (free text)
10. Why did you start your job search at a different time than recommended by your fellowship program? (free text)
11. What resources di you use to identify available jobs? (check all that apply)
    a. Cold emailing or calling programs
    b. Fellowship program leadership
    c. Job fairs
    d. Networking at national/society meetings/conferences
    e. Online job centers/mailing lists
    f. Physician recruiter
    g. Prior connections to other institutions
    h. Social connections (ie, NNPs, former fellows, residency classmates)
    i. Social media
    j. Other → what other resource(s) did you use to identify available jobs?
12. Please rank your top 3 most helpful resources (out of those chosen from above)

    a. Ranking matrix
13. Did you utilize the ONTPD/TECaN Job Search Series?
    a. Yes → Q15
    b. No → Q14
14. What was (were) the reason(s) for not utilizing the ONTPD/TECaN Job Search Series? (free text)
    a. → Q19
15. How did you utilize the Job Search Series? (check all that apply)
    a. Live session(s)
    b. Video recording(s)
16. For the following statements, choose true or false. Choose n/a if you did not attend/view the session on the topic.

    The Job Search Series was helpful in…

	True	False	N/A
Preparing my CV			
Preparing my cover letter			
Learning how to market myself			
Learning about contract negotiation			
Learning about how gender can affect contract negotiation			
Learning about the timeline of the job search			
Learning about the difference between "claims made-" and "occurrence-" liability insurance			
Learning about the roles and responsibilities of an academic faculty position			
Learning about the roles and responsibilities of a private practice faculty position			

Learning about typical starting salaries in my desired locations			
Learning how diversity, equity, and inclusion can affect the job search			
Learning about the transition from fellow to jr faculty			
Learning about job hunting with a VISA			

17. Were there any topics that came up during your job search that should be added to future ONTPD/TECaN Job Search Series?
    a. Yes → please elaborate (free text)
    b. No
18. Use the space below to write any additional comments regarding the ONTPD/TECaN Job Search Series. (free text)
19. How could a fellowship program best support fellows as they prepare for and engage in the job search? (check all that apply)
    a. Advocate for a position at your institution – if you were interested in staying
    b. Pass on information about jobs that may be of interest
    c. Pass on information about past fellows and their employment/contract information
    d. Provide a job search lecture/workshop
    e. Provide career/job search mentor(s)
    f. Provide mock interview(s)
    g. Reach out to personal contacts at other institutions
    h. Review job application material(s)
    i. Write a letter of recommendation for a job at another institution
    j. Other → please describe (free text)
20. Did you find the job search process during fellowship training stressful?
    a. Yes → Q21
    b. No → Q22
    c. N/A – I am not currently in fellowship
21. What made is stressful? (check all that apply)
    a. Competition with other applicants
    b. COVID restrictions
    c. Different timelines for different jobs
    d. I did not have a mentor that was knowledgeable in my career choice
    e. I did not have enough exposure to my preferred practice model

f. I did not know what to look for in a position
g. Limited job availability in preferred areas
h. Limited understanding of job search timeline and/or process
i. Minimal communication from job prospects
j. Personal or geographic constraints on my job search
k. Scheduling conflicts between fellowship clinical/research obligations and job interviews
l. There were not enough resources within my fellowship program to help guide my career plans
m. Other → please describe (free text)

22. Should job search preparation be structured into the fellowship curriculum?
    a. Yes → Q23
    b. No → Q24
23. When should this teaching occur? (check all that apply)
    a. 1st year of fellowship
    b. 2nd year of fellowship
    c. 3rd/4th year of fellowship
24. What have you found most challenging about your job search? (free text)
25. What did you wish you had known prior to your job search? (free text)
26. What tools or topics regarding the job search would be helpful for future trainees? (free text)

Finally, this last set of questions will help us better describe the specifics of job searchers within the field of neonatology and their outcomes. Your responses are anonymous and confidential. No personally identifiable information will be associated with your responses in any report of the data.

27. How many job positions did you apply for?
    a. Slider from 0 to > 9
    b. → Q28 if answer not equal 0
    c. If 0 → Q43
28. How many interview invitations did you receive?
    a. Slider from 0 to > 9
    b. → Q29 if answer not equal 0
    c. If 0 → Q32 and then Q43
29. How many of these interview invitations had an initial telephone screen?
    a. Slider from 0 to > 9
    b. → Q30 if answer not equal 0
30. Was the initial interview virtual or in person? (do not include any telephone screens)
    a. Virtual
    b. In person
    c. Hybrid
31. Have you accepted a job offer?
    a. Yes → Q33

b. No → Q32 then Q43

32. How long have you been actively looking for a job as a neonatologist?
    a. < 1 month
    b. 1-3 months
    c. 4-6 months
    d. 7-9 months
    e. 10-12 months
    f. 13-18 months
    g. > 18 months
33. How long did you actively look for a job as a neonatologist before accepting a position?
    a. < 1 month
    b. 1-3 months
    c. 4-6 months
    d. 7-9 months
    e. 10-12 months
    f. 13-18 months
    g. > 18 months
34. Have you signed a final contract?
    a. Yes → Q35
    b. No → Q36
35. In what month did you sign the contract for your new job? (drop box)
36. Did you consult with an attorney for contract negotiation assistance?
    a. Yes → Q37
    b. No → 38
37. Did you feel that the attorney provided benefit?
    a. Yes
    b. No → please explain (free text)
38. In retrospect, do you wish you had consulted with an attorney?
    a. Yes → please explain (free text)
    b. No
39. What area(s) of your contract did you negotiate? (check all that apply)
    a. I did not negotiate anything
    b. Bonus structure
    c. Call structure
    d. CME money
    e. Loan repayment
    f. Malpractice insurance
    g. Non-compete clause
    h. Professional development opportunities
    i. Protected research/educational time
    j. Relocation package
    k. Retirement package
    l. Salary

    m. Sign on bonus
    n. Start up package
    o. Vacation
    p. Other → free text
40. Were there any components of your contract that were non-negotiable?
    a. Yes → Q41
    b. No → Q42
41. What component(s) of your contract was (were) non-negotiable? (check all that apply)
    a. Bonus structure
    b. Call structure
    c. CME money
    d. Loan repayment
    e. Malpractice insurance
    f. Non-compete clause
    g. Professional development opportunities
    h. Protected research/educational time
    i. Relocation package
    j. Retirement package
    k. Salary
    l. Sign on bonus
    m. Start up package
    n. Vacation
    o. Other → free text
42. Please provide the space below to provide any additional detail regarding contract negotiation. (free text)
43. What is the single most importance piece of information that fellows should know to improve their success in applying for a job? (free text)
44. Please use the space below to provide any further comment on the job search process or ONTPD/TECaN's Job Search Series. (free text)
