# Supplementary material for: A national virtual job search series for neonatal-perinatal medicine fellows
Source: BMC Med Educ. 2024 Jun 6;24:633. doi: 10.1186/s12909-024-05587-9 (PMC11155180; doi:10.1186/s12909-024-05587-9)
Supplement: Supplementary file 3 — Supplementary Material 3 [file 12909_2024_5587_MOESM3_ESM.pdf]

Additional File 3: Categories of Answers Extracted from Survey Free-Text Questions

Box E1.

Most Challenging Aspects of the Job Search[a]

Process Issues

- **Navigating multiple timelines and the lack of a definitive timeline**
- **Identifying hiring institutions/practices; cold calling/emailing**
- Limited communication
- Finding the contact information for institutions/practices
- Balancing interviewing with clinical/fellowship duties
- Time-consuming and slow process of the job search
- Lack of transparency by programs in what they are looking for in a candidate
- Lack of networking
- No support for clinician-scientists without a K award

Personal Issues

- **Geographical limitations**
- Balancing partner vs own priorities; dual-career couple searches
- Lack of sufficient self-reflection of priorities prior to the job search
- Competition with co-fellows
- Job hunting with a visa
- Conflicting family and work priorities

Program/Mentorship Issues

- Choosing between very different positions
- Lack of knowledge of job search timeline and process
- Lack of mentors to go to for advice
- Lack of guidance from fellowship program
- How to compete with experienced candidates
- Lack of knowledge of what aspects of a job are most important to consider
- Fellowship program lack of information on private practice options

[a] Bold-type = top-3 answer

Box E2.

Topics Respondents Wish They Would Have Known Prior to the Job Search[a]

Process Topics

- **How time consuming and slow the job search process is**
- **How little or lack of communication from prospective employers there is**
- **How confusing the process is to find job openings or to identify NICUs in your desired area**
- **How different the timeline and process of the job search is compared to prior training stages**
- How to filter through jobs and identify job specifics/requirements before an interview
- How difficult it is to get protected time
- How competitive the academic market is
- How certain job openings are only looking for experienced neonatologists

Personal Topics

- **To start the job search process sooner**
- How to better market self
- How to better set personal priorities and self reflect prior to the job search
- How to craft a better cover letter
- How to craft a CV specific to the type of practice of interest
- How to schedule 3rd year clinical responsibilitiles to allow for interviewing
- To interview outside the fellowship institution even if a high likelihood of staying
- How to gauge whether a practice's or institution's practice patterns align with yours

Program/Mentorship Topics

- More information on the private practice job search
- The components of a contract and what is and is not usually negotiable
- Typical starting salaries
- Mock interviews
- How to structure answers to interview questions based on the practice model being pursued
- What a competitive offer looks like
- More information on hybrid practice models
- The importance of networking
- Distinguishing primary research positions from clinical positions
- How to gauge a practice's or institution's work culture
- How to gauge a practice's or institution's commitment to evidence-based practices

[a] Bold-type = top-5 answer

Box E3.

Recommended Topics for Future Job Search Series

Topics for Academic Positions	Topics for Both Academic and Private Practice Positions	Topics for Private Practice Positions
• How institutional budgeting and salaries work<br>• How grant funding works<br>• How to advocate for protected research time<br>• What the different tracks within academia are<br>• What the differences are between starting as an instructor vs assistant professor<br>• What FTEs are<br>• How best to divide up academic time<br>• Job talk tips	• More specifics on how to find job openings<br>• More specifics on the job search timeline<br>• More time dedicated to liability insurance<br>• Dual career household job searches<br>• How to assess work culture on the interview trail<br>• How to compete with experienced neonatologists<br>• How to prepare for the pre-interview phone call<br>• What components to think about to describe one's ideal job/practice<br>• How best to schedule 3rd year clinical/fellowship responsibilities<br>• What to include in an online profile<br>• How to approach gaps in training<br>• Interview tips	• How to maintain academic connections while in private practice<br>• More specifics on clinical loads and responsibilities<br>• More specifics on smaller private practices
